# (2*E*)-3-(2-Fluoro­phen­yl)-1-(4-fluoro­phen­yl)prop-2-en-1-one

**DOI:** 10.1107/S1600536812034411

**Published:** 2012-08-08

**Authors:** Hoong-Kun Fun, Abbas Farhadikoutenaei, B. Narayana, Prakash S. Nayak, B. K. Sarojini

**Affiliations:** a X-ray Crystallography Unit, School of Physics, Universiti Sains Malaysia, 11800 USM, Penang, Malaysia; b Department of Studies in Chemistry, Mangalore University, Mangalagangotri 574 199, India; c Department of Chemistry, P. A. College of Engineering, Nadupadavu, Montepadavu, PO, Mangalore 574 153, India

## Abstract

In the title compouund, C_15_H_10_F_2_O, the mol­ecule exists in an *E* conformation with respect to the C=C bond [1.3382 (16) Å]. The dihedral angle between the fluoro-substituted benzene rings is 6.80 (6)° and the whole mol­ecule is roughly planar (r.m.s. deviation for the non-H atoms = 0.069 Å). In the crystal, mol­ecules are linked by C—H⋯F and C—H⋯O inter­actions into sheets lying parallel to the *bc* plane.

## Related literature

For details of the synthesis of chalcones, see: Fun *et al.* (2012 ▶). For bond-length data, see: Allen *et al.* (1987 ▶). For the stability of the temperature controller used for data collection, see: Cosier & Glazer (1986 ▶).
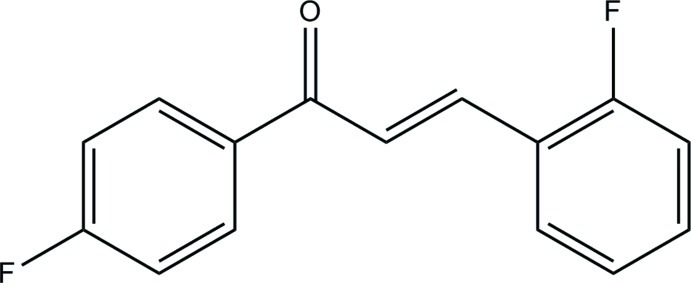


## Experimental

### 

#### Crystal data

C_15_H_10_F_2_O
*M* *_r_* = 244.23
Monoclinic, 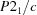

*a* = 14.569 (2) Å
*b* = 7.2737 (10) Å
*c* = 11.3933 (15) Å
β = 108.827 (3)°
*V* = 1142.7 (3) Å^3^
*Z* = 4
Mo *K*α radiation
μ = 0.11 mm^−1^
*T* = 100 K
0.25 × 0.23 × 0.10 mm

#### Data collection

Bruker APEX DUO CCD diffractometer
Absorption correction: multi-scan (*SADABS*; Bruker, 2009 ▶) *T*_min_ = 0.973, *T*_max_ = 0.989
12454 measured reflections
3316 independent reflections
2525 reflections with *I* > 2σ(*I*)
*R*_int_ = 0.031

#### Refinement

*R*[*F* ^2^ > 2σ(*F* ^2^)] = 0.042
*wR*(*F* ^2^) = 0.121
*S* = 1.02
3316 reflections
163 parameters
H-atom parameters constrained
Δρ_max_ = 0.34 e Å^−3^
Δρ_min_ = −0.27 e Å^−3^

### 

Data collection: *APEX2* (Bruker, 2009 ▶); cell refinement: *SAINT* (Bruker, 2009 ▶); data reduction: *SAINT*; program(s) used to solve structure: *SHELXTL* (Sheldrick, 2008 ▶); program(s) used to refine structure: *SHELXTL*; molecular graphics: *SHELXTL*; software used to prepare material for publication: *SHELXTL* and *PLATON* (Spek, 2009 ▶).

## Supplementary Material

Crystal structure: contains datablock(s) global, I. DOI: 10.1107/S1600536812034411/hb6921sup1.cif

Structure factors: contains datablock(s) I. DOI: 10.1107/S1600536812034411/hb6921Isup2.hkl

Supplementary material file. DOI: 10.1107/S1600536812034411/hb6921Isup3.cml

Additional supplementary materials:  crystallographic information; 3D view; checkCIF report

## Figures and Tables

**Table 1:** Hydrogen-bond geometry (Å, °)

*D*—H⋯*A*	*D*—H	H⋯*A*	*D*⋯*A*	*D*—H⋯*A*
C4—H4*A*⋯F2^i^	0.95	2.47	3.4145 (16)	175
C14—H14*A*⋯F1^ii^	0.95	2.54	3.4816 (16)	174
C15—H15*A*⋯O1^iii^	0.95	2.55	3.4956 (15)	174

Symmetry codes: (i) 
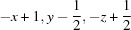
; (ii) 
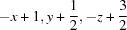
; (iii) 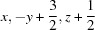.

**Fig. 1.**
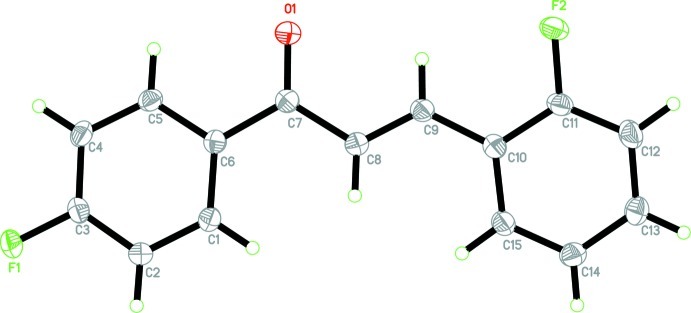
The molecular structure of the title compound, showing 50% probability displacement ellipsoids.

**Fig. 2.**
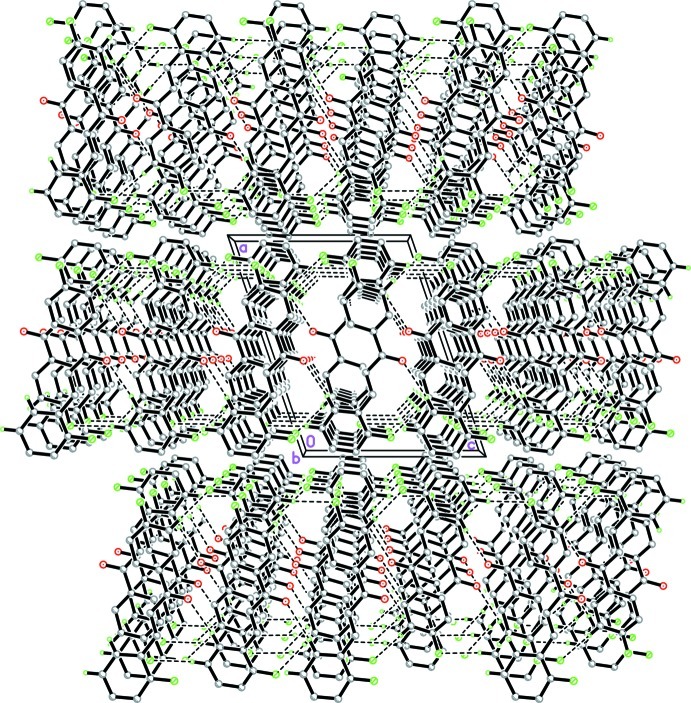
The crystal packing of the title compound, viewed along the b axis.

## supplementary crystallographic information

### Comment

In continuation of our work on synthesis of chalcone derivatives (Fun *et al.*, 2012), the title compound (I) has been prepared and its crystal structure is now reported.

In the title compouund (Fig. 1), the molecule exists in an *E* conformation with respect to C8═C9 [1.3382 (16) Å]. The dihedral angle between the fluoro-substituted (C1–C6 & C10–C15) benzene rings is 6.80 (6)°. The bond lengths (Allen *et al.*, 1987) and angles are within normal ranges.

In the crystal structure (Fig. 2), the molecules are linked *via* C4—H4A···F2, C14—H14A···F1 and C15—H15A···O1 hydrogen bonds (Table 1) into two dimensional networks parallel to *bc* plane.

### Experimental

To a mixture of 4-fluoroacetophenone (1.38 g, 0.01 mol) and 2-fluorobenzaldehyde (1.05 ml, 0.01 mol) in ethanol (100 ml), 15 ml of 10% sodium hydroxide solution was added and stirred at 0–5 °C for 3 h. The precipitate formed was collected by filtration and purified by recrystallization from ethanol. Colourless blocks were grown from methanol solution by the slow evaporation method (*M.P.*: 351 K).

### Refinement

All the H atoms were positioned geometrically and refined using a riding model with *U*_iso_(H) = 1.2 *U*_eq_(C) (C—H = 0.95 Å).

### Crystal data

**Table:**

C_15_H_10_F_2_O	*F*(000) = 504
*M**_r_* = 244.23	*D*_x_ = 1.420 Mg m^−^^3^
Monoclinic, *P*2_1_/*c*	Mo *K*α radiation, λ = 0.71073 Å
Hall symbol: -P 2ybc	Cell parameters from 3240 reflections
*a* = 14.569 (2) Å	θ = 3.0–30.0°
*b* = 7.2737 (10) Å	µ = 0.11 mm^−^^1^
*c* = 11.3933 (15) Å	*T* = 100 K
β = 108.827 (3)°	Block, colourless
*V* = 1142.7 (3) Å^3^	0.25 × 0.23 × 0.10 mm
*Z* = 4	

### Data collection

**Table:**

Bruker APEX DUO CCD diffractometer	3316 independent reflections
Radiation source: fine-focus sealed tube	2525 reflections with *I* > 2σ(*I*)
Graphite monochromator	*R*_int_ = 0.031
φ and ω scans	θ_max_ = 30.1°, θ_min_ = 1.5°
Absorption correction: multi-scan (*SADABS*; Bruker, 2009)	*h* = −20→19
*T*_min_ = 0.973, *T*_max_ = 0.989	*k* = −8→10
12454 measured reflections	*l* = −14→16

### Refinement

**Table:**

Refinement on *F*^2^	Primary atom site location: structure-invariant direct methods
Least-squares matrix: full	Secondary atom site location: difference Fourier map
*R*[*F*^2^ > 2σ(*F*^2^)] = 0.042	Hydrogen site location: inferred from neighbouring sites
*wR*(*F*^2^) = 0.121	H-atom parameters constrained
*S* = 1.02	*w* = 1/[σ^2^(*F*_o_^2^) + (0.0556*P*)^2^ + 0.4256*P*] where *P* = (*F*_o_^2^ + 2*F*_c_^2^)/3
3316 reflections	(Δ/σ)_max_ < 0.001
163 parameters	Δρ_max_ = 0.34 e Å^−^^3^
0 restraints	Δρ_min_ = −0.27 e Å^−^^3^

### Special details

**Table:**

Experimental. The crystal was placed in the cold stream of an Oxford Cryosystems Cobra open-flow nitrogen cryostat (Cosier & Glazer, 1986) operating at 100.0 (1) K.
Geometry. All e.s.d.'s (except the e.s.d. in the dihedral angle between two l.s. planes) are estimated using the full covariance matrix. The cell e.s.d.'s are taken into account individually in the estimation of e.s.d.'s in distances, angles and torsion angles; correlations between e.s.d.'s in cell parameters are only used when they are defined by crystal symmetry. An approximate (isotropic) treatment of cell e.s.d.'s is used for estimating e.s.d.'s involving l.s. planes.
Refinement. Refinement of *F*^2^ against ALL reflections. The weighted *R*-factor *wR* and goodness of fit *S* are based on *F*^2^, conventional *R*-factors *R* are based on *F*, with *F* set to zero for negative *F*^2^. The threshold expression of *F*^2^ > σ(*F*^2^) is used only for calculating *R*-factors(gt) *etc*. and is not relevant to the choice of reflections for refinement. *R*-factors based on *F*^2^ are statistically about twice as large as those based on *F*, and *R*- factors based on ALL data will be even larger.

### Fractional atomic coordinates and isotropic or equivalent isotropic displacement parameters (Å^2^)

**Table:**

	*x*	*y*	*z*	*U*_iso_*/*U*_eq_	
F1	0.16014 (5)	0.57105 (12)	0.38021 (7)	0.0270 (2)	
F2	0.88539 (6)	0.90022 (15)	0.48557 (7)	0.0357 (2)	
O1	0.56195 (6)	0.67338 (14)	0.29627 (8)	0.0249 (2)	
C1	0.40989 (8)	0.71839 (17)	0.49338 (11)	0.0190 (2)	
H1A	0.4546	0.7712	0.5656	0.023*	
C2	0.31519 (9)	0.68328 (18)	0.48962 (11)	0.0203 (2)	
H2A	0.2945	0.7111	0.5586	0.024*	
C3	0.25209 (8)	0.60721 (17)	0.38349 (11)	0.0195 (2)	
C4	0.27869 (9)	0.56238 (18)	0.28129 (11)	0.0209 (3)	
H4A	0.2334	0.5097	0.2096	0.025*	
C5	0.37332 (9)	0.59650 (18)	0.28640 (11)	0.0193 (2)	
H5A	0.3935	0.5653	0.2176	0.023*	
C6	0.43978 (8)	0.67640 (16)	0.39157 (11)	0.0171 (2)	
C7	0.54041 (8)	0.71165 (17)	0.38906 (11)	0.0181 (2)	
C8	0.61241 (8)	0.79265 (18)	0.50023 (11)	0.0198 (2)	
H8A	0.5931	0.8268	0.5693	0.024*	
C9	0.70435 (8)	0.81809 (17)	0.50426 (11)	0.0189 (2)	
H9A	0.7203	0.7847	0.4326	0.023*	
C10	0.78236 (8)	0.89267 (17)	0.60915 (11)	0.0183 (2)	
C11	0.87245 (9)	0.93383 (19)	0.59684 (11)	0.0222 (3)	
C12	0.94940 (9)	1.0061 (2)	0.69058 (13)	0.0264 (3)	
H12A	1.0091	1.0334	0.6773	0.032*	
C13	0.93744 (9)	1.0379 (2)	0.80465 (12)	0.0256 (3)	
H13A	0.9893	1.0873	0.8710	0.031*	
C14	0.84936 (9)	0.9974 (2)	0.82199 (12)	0.0252 (3)	
H14A	0.8416	1.0181	0.9006	0.030*	
C15	0.77291 (9)	0.92723 (18)	0.72577 (12)	0.0215 (3)	
H15A	0.7130	0.9021	0.7390	0.026*	

### Atomic displacement parameters (Å^2^)

**Table:**

	*U*^11^	*U*^22^	*U*^33^	*U*^12^	*U*^13^	*U*^23^
F1	0.0162 (3)	0.0386 (5)	0.0260 (4)	−0.0048 (3)	0.0066 (3)	−0.0005 (3)
F2	0.0216 (4)	0.0672 (7)	0.0203 (4)	−0.0037 (4)	0.0097 (3)	−0.0020 (4)
O1	0.0216 (4)	0.0347 (5)	0.0197 (4)	−0.0006 (4)	0.0083 (3)	−0.0016 (4)
C1	0.0180 (5)	0.0196 (6)	0.0180 (5)	−0.0003 (4)	0.0037 (4)	−0.0008 (5)
C2	0.0196 (5)	0.0232 (6)	0.0184 (6)	0.0001 (5)	0.0068 (4)	−0.0013 (5)
C3	0.0156 (5)	0.0208 (6)	0.0215 (6)	−0.0006 (4)	0.0052 (4)	0.0033 (5)
C4	0.0199 (5)	0.0233 (6)	0.0166 (5)	−0.0022 (5)	0.0019 (4)	0.0014 (5)
C5	0.0209 (5)	0.0213 (6)	0.0151 (5)	0.0001 (4)	0.0048 (4)	0.0009 (4)
C6	0.0167 (5)	0.0164 (5)	0.0173 (5)	0.0015 (4)	0.0042 (4)	0.0018 (4)
C7	0.0180 (5)	0.0181 (6)	0.0176 (5)	0.0015 (4)	0.0050 (4)	0.0028 (4)
C8	0.0192 (5)	0.0220 (6)	0.0180 (5)	−0.0003 (4)	0.0056 (4)	−0.0003 (5)
C9	0.0185 (5)	0.0213 (6)	0.0166 (5)	0.0012 (4)	0.0052 (4)	0.0022 (4)
C10	0.0164 (5)	0.0195 (6)	0.0185 (5)	0.0018 (4)	0.0048 (4)	0.0037 (5)
C11	0.0184 (5)	0.0316 (7)	0.0172 (5)	0.0021 (5)	0.0067 (4)	0.0032 (5)
C12	0.0152 (5)	0.0373 (8)	0.0255 (6)	−0.0016 (5)	0.0047 (5)	0.0030 (6)
C13	0.0193 (6)	0.0308 (7)	0.0230 (6)	−0.0013 (5)	0.0019 (5)	−0.0008 (5)
C14	0.0230 (6)	0.0318 (7)	0.0200 (6)	0.0007 (5)	0.0061 (5)	−0.0025 (5)
C15	0.0179 (5)	0.0260 (6)	0.0210 (6)	−0.0003 (5)	0.0068 (4)	0.0008 (5)

### Geometric parameters (Å, º)

**Table:**

F1—C3	1.3539 (13)	C8—C9	1.3382 (16)
F2—C11	1.3627 (14)	C8—H8A	0.9500
O1—C7	1.2279 (15)	C9—C10	1.4621 (17)
C1—C2	1.3901 (16)	C9—H9A	0.9500
C1—C6	1.3980 (16)	C10—C11	1.3966 (16)
C1—H1A	0.9500	C10—C15	1.4021 (17)
C2—C3	1.3769 (17)	C11—C12	1.3788 (18)
C2—H2A	0.9500	C12—C13	1.3852 (19)
C3—C4	1.3797 (17)	C12—H12A	0.9500
C4—C5	1.3834 (17)	C13—C14	1.3915 (18)
C4—H4A	0.9500	C13—H13A	0.9500
C5—C6	1.4007 (16)	C14—C15	1.3837 (18)
C5—H5A	0.9500	C14—H14A	0.9500
C6—C7	1.4977 (16)	C15—H15A	0.9500
C7—C8	1.4813 (17)		
			
C2—C1—C6	120.40 (11)	C7—C8—H8A	119.7
C2—C1—H1A	119.8	C8—C9—C10	125.79 (11)
C6—C1—H1A	119.8	C8—C9—H9A	117.1
C3—C2—C1	118.40 (11)	C10—C9—H9A	117.1
C3—C2—H2A	120.8	C11—C10—C15	116.08 (11)
C1—C2—H2A	120.8	C11—C10—C9	120.35 (11)
F1—C3—C2	118.46 (11)	C15—C10—C9	123.57 (10)
F1—C3—C4	118.40 (11)	F2—C11—C12	117.84 (11)
C2—C3—C4	123.12 (11)	F2—C11—C10	118.14 (11)
C3—C4—C5	118.01 (11)	C12—C11—C10	124.02 (11)
C3—C4—H4A	121.0	C11—C12—C13	118.25 (11)
C5—C4—H4A	121.0	C11—C12—H12A	120.9
C4—C5—C6	120.98 (11)	C13—C12—H12A	120.9
C4—C5—H5A	119.5	C12—C13—C14	119.92 (12)
C6—C5—H5A	119.5	C12—C13—H13A	120.0
C1—C6—C5	119.08 (11)	C14—C13—H13A	120.0
C1—C6—C7	123.19 (10)	C15—C14—C13	120.60 (12)
C5—C6—C7	117.73 (10)	C15—C14—H14A	119.7
O1—C7—C8	121.28 (11)	C13—C14—H14A	119.7
O1—C7—C6	120.09 (11)	C14—C15—C10	121.11 (11)
C8—C7—C6	118.63 (10)	C14—C15—H15A	119.4
C9—C8—C7	120.60 (11)	C10—C15—H15A	119.4
C9—C8—H8A	119.7		
			
C6—C1—C2—C3	−0.23 (19)	C6—C7—C8—C9	176.72 (11)
C1—C2—C3—F1	179.28 (11)	C7—C8—C9—C10	−178.59 (11)
C1—C2—C3—C4	0.7 (2)	C8—C9—C10—C11	−172.08 (13)
F1—C3—C4—C5	−178.75 (11)	C8—C9—C10—C15	7.9 (2)
C2—C3—C4—C5	−0.2 (2)	C15—C10—C11—F2	179.16 (11)
C3—C4—C5—C6	−0.83 (19)	C9—C10—C11—F2	−0.85 (19)
C2—C1—C6—C5	−0.76 (18)	C15—C10—C11—C12	−0.7 (2)
C2—C1—C6—C7	179.61 (11)	C9—C10—C11—C12	179.33 (13)
C4—C5—C6—C1	1.31 (18)	F2—C11—C12—C13	−178.95 (12)
C4—C5—C6—C7	−179.04 (11)	C10—C11—C12—C13	0.9 (2)
C1—C6—C7—O1	−179.26 (12)	C11—C12—C13—C14	−0.2 (2)
C5—C6—C7—O1	1.10 (17)	C12—C13—C14—C15	−0.7 (2)
C1—C6—C7—C8	0.84 (18)	C13—C14—C15—C10	0.9 (2)
C5—C6—C7—C8	−178.80 (11)	C11—C10—C15—C14	−0.25 (19)
O1—C7—C8—C9	−3.18 (19)	C9—C10—C15—C14	179.76 (12)

### Hydrogen-bond geometry (Å, º)

**Table:**

*D*—H···*A*	*D*—H	H···*A*	*D*···*A*	*D*—H···*A*
C4—H4*A*···F2^i^	0.95	2.47	3.4145 (16)	175
C14—H14*A*···F1^ii^	0.95	2.54	3.4816 (16)	174
C15—H15*A*···O1^iii^	0.95	2.55	3.4956 (15)	174

Symmetry codes: (i) −*x*+1, *y*−1/2, −*z*+1/2; (ii) −*x*+1, *y*+1/2, −*z*+3/2; (iii) *x*, −*y*+3/2, *z*+1/2.
